# Long-term consequences of sexually transmitted infections on men’s sexual function: A systematic review

**DOI:** 10.1080/2090598X.2021.1942414

**Published:** 2021-07-07

**Authors:** Ralf Henkel

**Affiliations:** a Department of Metabolism, Digestion and Reproduction, Imperial College London, London, UK; b Department of Medical Bioscience, University of the Western Cape, Bellville, South Africa; c Department of Urology, LogixX Pharma, Theale, Reading, UK; d American Center for Reproductive Medicine, Cleveland Clinic, Cleveland, OH, USA

**Keywords:** Sexually transmitted diseases, long-term effects, male infertility, genital tract infection, obstruction

## Abstract

**Objective**: To systematically review the available literature on the long-term effects of sexually transmitted diseases (STIs) on male reproductive functions.

**Methods:** A PubMed search was conducted on 3 January 2021, and as a result, 952 articles were retrieved. Exclusion of irrelevant articles resulted in 36 articles, dating from 1998 to 2020, which were analysed.

**Results:** Only 52.8% of these articles described original research, while the rest were reviews. The majority (26) of the articles dealt with bacterial infections, of which 20 described *Chlamydia trachomatis*. There were 11 articles that described research on viruses, with five on severe acute respiratory syndrome coronavirus 2 (SARS-CoV-2). The analysis of the articles showed further that not much new knowledge on the long-term effects on male reproductive functions has been added. The existing knowledge that ascending infections can cause epididymo-orchitis, prostatitis or urethritis was confirmed. Due to epithelial inflammatory responses these infections can result in scarring with resulting infertility due to obstruction. These effects were described for *Chlamydia trachomatis, Neisseria gonorrhoeae, Mycoplasma genitalium* or *Treponema pallidum*, as well as for the Zika and SARS-CoV-2 viruses. Even trichomoniasis can lead to long-term compromised male fertility if not treated.

**Conclusion:** In conclusion, problem awareness needs to be raised and more research on this important topic needs to be conducted.

## Introduction

Infertility is a globally underestimated public health issue [1] with significant socioeconomic and psychological impact on both male and female partners. On average ~15% of couples of reproductive age are infertile [2], amounting to almost 200 million couples with a percentage contribution of the male ranging between 20% and 70% [3]. Among the causes of male infertility, genital tract infections are the third most common (11.6%), with some authors even reporting up to 35% [4–6]. Both, acute and chronic male genital tract infections can cause or significantly contribute to temporary male fertility problems. As most of the genital tract infections are sexually transmittable and very often asymptomatic, a male genital tract infection does not only cause male reproductive dysfunctions but can also lead to female infertility and even problems or diseases in the offspring.

Sexually transmitted diseases are a major public health issue with >1 million people acquiring a sexually transmitted infection (STI) every day [7]. Out of the eight most prevalent STIs (chlamydia, gonorrhoea, syphilis, trichomoniasis, hepatitis B virus [HBV], herpes, HIV, and human papilloma virus [HPV]), four are curable (chlamydia, gonorrhoea, syphilis, and trichomoniasis). Yet, and despite of the sexual transmissibility, the public awareness and research efforts are rather focussed on women because the burden of the disease sequelae with pregnancy complication etc. are considered a female problem [8]. Nevertheless, men are equally affected by STIs and should therefore garner the same attention. These STIs lead to manifold problems including discomfort and burning sensations while urinating or having intercourse, up to severe pain of the testicles. Long-term effects, such as infertility due to testicular damage or scarring of the efferent seminal ducts, have been described. As summarised information about the long-term effects of STIs on male reproductive functions is missing, the aim of the present review was to summarise updated information on this important issue.

## Methods

**Figure 1.**
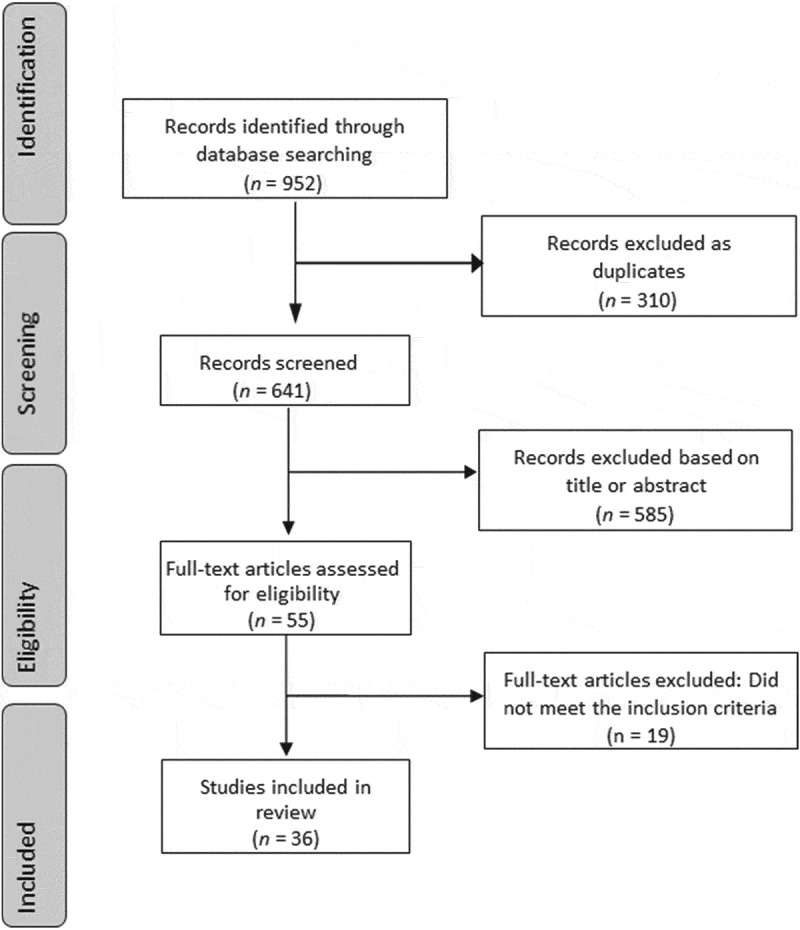
PRISMA flow chart of the literature search with selection process

A literature search on Medline (PubMed), using the search strings shown in Supplementary Table S1, was conducted according to the Preferred Reporting Items for Systematic Reviews and Meta-Analyses (PRISMA) guidelines [9]. The search was conducted on 3 January 2021 and had no time or language restriction and included original research articles, case reports, and reviews. Further article selection was done according to the title and afterwards based on the information provided in the abstract. Thus, only articles focussing on human male fertility and the effects of sexually transmitted diseases/STIs were included in further analyses. In contrast, articles dealing with e.g. social aspects, technical aspects, animal species, or prevention of STIs in women etc., were excluded. Subsequently, the full text of the article was reviewed where then additional information was obtained. The search and selection process are shown in Figure 1.

## Results

A total of 952 articles were retrieved from the PubMed search, of which 310 articles appeared in different searches and were then excluded. The remaining 642 articles were checked for whether their titles matched the topic. This selection led to the elimination of 587 articles. Eventually, the abstracts of 55 articles were analysed and a further 19 articles eliminated because they were not relevant, leaving a total of 36 articles for full analysis, with 17 reviews and 19 original articles. All these articles were written in the English language. Figure 1 depicts the search strategy with the selection process of the articles that were eventually analysed. The articles were published between 1998 and 2020, with 26 articles published in the past 10 years. A clear trend reflecting the current pandemic of severe acute respiratory syndrome coronavirus 2 (SARS-CoV-2) could also be seen, with five articles out of seven published in 2020. Table 1 shows the specifications of the analysed studies [8,10–44].

**Table 1.** List of articles identified by the PubMed search shown according to ‘year of publication’, ‘type of article’, ‘pathogen species’ and ‘type of pathogen’

	Articles, *n*	Reference
**Year of publication**		
1998	1	[23]
1999	1	[29]
2001	1	[30]
2002	1	[32]
2003	2	[33,34]
2008	2	[8,24]
2009	1	[25]
2012	2	[35,36]
2013	2	[10,28]
2014	2	[15,37]
2016	4	[16,27,38,39]
2017	4	[11,12,31,40]
2018	4	[13,26,41,42]
2019	2	[14,43]
2020	7	[17–22,44]
Total	36	
**Type of article**		
Review	17	[8,10–12,15,16,18–23,27–29,34,44]
Original article	19	[13,14,17,24–26,30–33,35–43]
Total	36	
**Bacteria**		
**Pathogen species**		
*Chlamydia trachomatis* and related	20	[8,12–17,23–26,28,29,32,35,36,38,41,43,44]
*Neisseria*	7	[13,16,28,36,38,40,41]
*Mycoplasma*	5	[28,31,35,41,43]
*Ureaplasma*	5	[23,28,30,35,41]
*Treponema pallidum*	4	[16,33,36,40]
*Escherichia coli*	2	[28,42]
*Staphylococcus aureus*	1	[42]
*Streptococcus*	1	[42]
**Protozoa**		
*Trichomonas vaginalis*	5	[27,31,38,40,41]
**Viruses**		
SARS-CoV-2	5	[18–20,22]
HIV	3	[10,23,44]
HPV	5	[10,23,34,37,44]
ZIKV	1	[11]
HSV	3	[10,23,28]
HBV	1	[10]
HCV	1	[10]
HCMV	1	[10]
AAV	1	[10]
**Fungi**		
*Candida spp.*	1	[40]
**Type of pathogen**		
Bacteria	26	[8,12–17,23–34,36,38,40–44]
Viruses	11	[10,11,18–23,36,37,44]
Fungi	1	[40]
Protozoa	5	[27,31,38,40,41]

The overall number of the retrieved articles reflects the global number of infections with the relevant pathogen and their importance. As chlamydial infections are the most prevalent sexually transmitted disease, as expected, the majority (*n*= 20) of the articles dealt with *Chlamydia trachomatis* or related pathogens, followed by *Neisseria gonorrhoeae* (*n* = 7), *Mycoplasma spp*. (*n* = 5) and *Ureaplasma spp*. (*n* = 5). Other pathogens were *Treponema pallidum, Escherichia coli, Staphylococcus aureus* and *Streptococcus spec*. Five articles investigated the protozoon *Trichomonas vaginalis*. In the group of the viruses, SARS-CoV-2 and HPV were most investigated (*n* = 5), followed by herpes simplex virus (HSV; *n* = 3), whereas all other viruses (Zika virus [ZIKV], HBV, hepatitis C virus [HCV], cytomegalovirus [HCMV] and adeno-associated virus [AAV]) were only mentioned in the article. The fungus *Candida spp*. was mentioned in one article.

While most of the articles discuss the general effects of sexually transmitted male genital tract infections, only five articles [10–14] dealt with the long-lasting effects of the infection. Three of these articles discussed chlamydial infections [12–14] and two various viruses [10,11]. In the male, depending on the pathogen, the health issues reported ranged from chronic prostatitis, vesiculitis or epididymo-orchitis, leading to hormonal imbalances and testicular dysfunction, with disturbed spermatogenesis eventually resulting in temporary or permanent infertility. Generally, especially for chlamydia infections, however, many articles highlighted the fact that these infections are often silent, i.e. asymptomatic, and are therefore not or only at a late stage treated.

While early articles rather discussed the sites of infection, the type of disease and the relevant treatment options, they did not elaborate on long-term effects and the consequences of the infection. Only more recent articles discussed long-term effects such as the development of immunity due to bacterial infections specifically with *C. trachomatis* [8,12,15–17], which might be a reason why a high percentage of patients are asymptomatic and it is difficult to diagnose this infection, the effects of these viruses on the male reproductive system are investigated. Moreover, as a result of the 2016/2017 infection wave in Brazil and the USA with the ZIKV [8], and even more so because of the current coronavirus disease 2019 (COVID-19) pandemic [18], articles elaborated on the immune response to these viral infections.

## Discussion

In general, the present review found that the number of original articles investigating the long-term effects of STIs on male reproductive functions was incredibly low; almost 50% of the articles found were reviews. If one then further considers that various pathogens are causing STIs, not many individual articles on the specific effects of specific pathogens have been published. This reflects the general perception that not only reproduction, but also fertility problems and knowledge, are considered a female issue [8]. The fact that the diseases are asymptomatic in many cases exacerbates this problem because the patient him/herself does not realise they have the illness and thereby infect their sexual partners. Further, as sexual intercourse involves not only one person, but two, one should not only think of one individual when thinking of long-term consequences of sexually transmitted diseases in the male, because the long-term consequences can very well take place in the female where inflammatory processes can lead to tubal infertility through scarring or pregnancy complications. If not treated, it can eventually even cause illness in the offspring.

Even though the highest number of articles was published in 2020, this does not reflect an increased awareness of the importance of male genital tract infections in general and their consequences for health and the success of reproductive efforts but is rather due to the current COVID-19 pandemic with its research interest in SARS-CoV-2; five [18–22] out of seven articles published in 2020 were on SARS-CoV-2.

### Bacterial infections

Male genital tract infections, like male infertility in general, are undoubtedly not only an underestimated and increasing public health issue, but also an under-researched issue. This is due to several reasons, namely male self-image and self-esteem, humiliation, and lack of knowledge about reproduction as men think reproduction is a female issue [45–47]; hence, where their fertility is concerned, men are rather quiet as compared to women who are more open to these issues. Moreover, as major research efforts, screening and treatment are rather focussed on female and children’s health as sequelae of the problem, infertility and specifically problems around sexually transmitted diseases are regarded as a female problem [8]. Yet, the latter argument is not only true for STIs, but for male infertility issues in general leading to the fact that male infertility and its causes, despite the progress made, due to ignorance, is still a ‘hidden’ reproductive health condition [48].

In the present study it became obvious that most of the articles published deal with bacterial infections, specifically with *C. trachomatis*, which involved 72% and 55.5% of the analysed articles, respectively, and is thus reflecting the fact that chlamydial infections are recognised as the most prevalent bacterial infection, with a global prevalence of 3.8% in women and 2.7% in men. The total incidence is estimated at 127.2 million [49]. Overall, the asymptomatic courses of the infection of 75% in women and 50% in men [8] will even be exacerbated by the fact that the rate in young men, which are the sexually most active group, is significantly higher than in older men [50]. In addition, it has been shown that the testing rate in men is significantly lower than in women [51], leaving this group of men underdiagnosed, thus resulting in a high risk of infecting/re-infecting the female partner. These effects can then not be regarded as direct long-term effects, but rather as indirect effects such as complications of pregnancy, miscarriage, preterm labour and stillbirth associated with tubal infertility. Insofar, even though pregnancy complications are not direct long-term consequences of male genital tract infections, they should rather be seen as indirect consequences of an undetected infection, because of the asymptomatic nature and untreated genital tract infections.

In the male, consequences of chlamydial or chlamydia-related infections such as orchitis, epididymo-orchitis, prostatitis leading to infertility, particularly in acute cases of epididymo-orchitis [52], have been described, although debated [23–26,53]. Gaydos [54] describes that after Chlamydia infections no long-term immunity develops and that long-term male infertility would only occur after recurrent inflammations causing tissue damage. A recent *in vitro* study revealed that *C. trachomatis* can infect and replicate in primary human Sertoli cells, thus indicating that this bacterium may compromise the human blood–testis barrier [55]. Such damage could not only lead to impairment of spermatogenesis, but also disrupt the innate immune response of Sertoli cells by down-regulating nuclear factor kappa B (NF-κB) and interferon regulatory factor 3 (IRF3)-dependent pathways leading to the non-production of pro-inflammatory cytokines interleukin (IL)-1α, IL-6, interferon (IFN)-α, IFN-β and IFN-γ [17]. The active agents in these processes seem to be the chlamydial lipopolysaccharides that stimulate toll-like receptor-4 and a stimulator of IFN genes that results in NF-κB activation [56], thereby triggering an innate immune response [57]. However, after persistent infection, *C. trachomatis* can survive the immune attack by switching its metabolism into a replicative viable state by evading the immune response and seizing the host cells for its purposes, a process that can be seen in infections becoming chronic [58,59]. Such processes could then be responsible for endothelial dysfunction, fibrosis, scarring and obstruction of the reproductive tract resulting in infertility. Apart from chronic prostatitis and infertility, urethral strictures following chlamydial infections have been described [60]. These processes would then exacerbate repeated and/or chronic infections/inflammatory processes leading to a permanent damage. In animal studies it was shown that the chlamydial infection caused a significant loss of germ cells due to spermatogonial apoptosis leading to the most affected seminiferous tubules containing only Sertoli cells, also with increased apoptotic activity [61].

It also appears that *C. trachomatis* is directly affecting sperm function by having direct negative effects on spermatozoa, e.g. by causing sperm DNA fragmentation [62], a finding which could be due to increased seminal oxidative stress because of significantly increased seminal levels of leucocytes and pro-inflammatory cytokines IL-6 and IL-8 [63]. The active compounds causing this damage appears to be lipid A, a component of the lipopolysaccharides of *C. trachomatis* elementary bodies [64]. However, as for chlamydial infections effective treatment options are available and the disease is therefore potentially preventable, it generates less attention, although it has a significant morbidity. Yet, better prevention means that more screening and research should be conducted, so that a greater awareness can be achieved, specifically in the man.

For *Neisseria gonorrhoeae*, which is with 86.9 million cases the second most common sexually transmitted bacterial disease [49], a urethritis is most frequently reported. In ~10% of the cases, the disease is asymptomatic. However, it can also cause vesiculitis, penile oedema, prostatitis, orchitis or chronic epididymitis if the disease is untreated [65]. Acute epididymo-orchitis often results in poor semen quality with a decreased sperm count and motility [66]. Furthermore, ascending infections can have, if not properly treated, long-term effects such as testicular damage or azoospermia due to obstruction [67]. Another problem that will exacerbate this issue in the long term is the increasing multidrug resistance of this pathogen.

As for chlamydial infections, the expression levels of IL-1 and IL-8 have a direct effect on the development and severity of a urethritis after infection with *N. gonorrhoeae* as shown by the odds ratios in the study by Singer and Ouburg [16]. The strongest association between the severity of the inflammatory response was found for the IL-1 levels, whereas IL-8 caused a lesser response, also in terms of complications of the infection, thus indicating that the innate immune system with the participation of NF-κB activated cytokines is the predominantly involved in the *N. gonorrhoeae* infection and development of complications [16,68]. Such immune responses probably influencing the epithelial inflammatory response responsible for scarring and resulting in infertility due to obstructions can also be seen after infections with other pathogens such as *C. trachomatis, M. genitalium* or *T. pallidum*.

### Protozoa

*Trichomonas vaginalis* is an anaerobic protozoal parasite that with an estimated 248 million new infections was regarded by the WHO as the most prevalent sexually transmitted disease in 1999 [69]. In men, trichomoniasis can cause urethritis, which is symptomatic in only ~30% of the cases. However, if the infection is ascending, it can also cause prostatitis or epididymitis [70]. While in women, the infection is frequently linked with pregnancy complications [71], an *in vitro* study by Tuttle *et al*. [72] showed a dramatic decrease in sperm motility as direct effect of the pathogen on sperm. A study by Gopalkrishnan *et al*. [73] not only indicated a significant decrease in sperm motility, but also in viability and normal sperm morphology. Although the impact of a trichomonal infection on male fertility potential is still controversially discussed, Ozdemir *et al*. [74] indicate that it should be considered for the aetiology of male factor infertility. In a case study, Lucena *et al*. [75] reported the assisted reproduction of a couple with a *T. vaginalis*-positive asthenozoospermic semen sample. Fertilisation with capacitated sperm in *in vitro* fertilisation was achieved, but implantation failed, possibly due to the asymptomatic female. Considering this undeniable risk of male consequences of trichomonal infections, Mielczarek and Blaszkowska [27] reported that point-of-care tests have been developed in the female, but not evaluated in the male. In addition, apart from the pregnancy complications, trichomoniasis in the man may lead to long-term problems such as infertility or even prostate cancer, for which an association has been shown [76].

### Viral infections

The ZIKV has long been considered harmless. However, in recent years evidence emerged that apart from seriously affecting pregnancy outcomes, it has been found in high concentrations in the testes and epididymis in animal studies [77] and can actively reproduce in the male reproductive organs, thereby causing acute orchitis in a mouse model [11]. Considering that ZIKV specifically infects spermatogonia, primary spermatocytes and Sertoli cells, this will lead to permanent damage of the seminiferous tubules resulting in long-term infertility. In addition, decreased testosterone and inhibin B levels were observed [78], indicating possible Leydig and Sertoli cell damage. In addition, ZIKV has been reported to persist in semen with intermittent shedding [79], even in patients with non-obstructive azoospermia [80], hence indicating that the virus is either associated with leucocytes or epithelial cells or is freely available in the seminal fluid. As no orchitis has been reported in the human to date, the risk of obstruction and subsequent infertility due to ZIKV appears to be low.

The HPV is mostly known for pregnancy complications and cervical cancer as a leading cause of death in young women [81]. However, there is also a high prevalence of HPV infections in men with the spermatozoa apparently being a vector for infecting the female partner during intercourse [82]. It seems that the virus reduces sperm motility and increases sperm DNA fragmentation [82,83]. As the infection is often asymptomatic, many couples will only be identified as being infected when they fail to conceive. However, the possible long-term effects on male reproductive function need further investigation.

SARS-CoV-2 is a virus that emerged from Wuhan, China, in December 2019 causing the COVID-19 pandemic. As cell entry of the virus is mediated by angiotensin converting enzyme-2 (ACE2) and this receptor protein is highly expressed in Leydig and Sertoli cells, as well as in spermatogonia and seminiferous tubules, SARS-CoV-2 can infect the male reproductive system, indicating the potential risk that sexual transmission may be possible [84]. However, to date, there has been no positive report of a sexual transmission and a recent review of the currently available literature [85] indicated that SARS-CoV-2 is not sexually transmitted. Nevertheless, at this moment, it is not clear whether illness with COVID-19 will cause long-term damage to spermatogenesis and/or its endocrine regulation. At least one can expect an impaired sperm production with poor sperm motility due to testicular heat stress during periods of fever [86] or decreased testosterone levels due to Leydig cell damage [87].

A limitation of the present study could be that the search resulted in almost 50% of the articles being a review. In addition, the present search was mainly focussed on human studies, whereas much of the basic work is done in animals, which had to be referred to in the discussion. This is also reflected by the fact that many of the articles were then retrieved by studying the selected articles. On the other hand, by focussing on human studies this clearly shows the paucity of research done on the long-term effects of STIs on male reproductive functions.

## Conclusion

Sexually transmitted diseases are serious illnesses, for which they are reportable. These diseases do not only have serious health consequences for the infected person, but also for the sexual partner and even for the possible offspring, if a conception takes place, an effect, which can, and should be, considered as late and long-term effect of an STI in the male. On the other hand, an ascending male genital tract infection can lead to long-term male infertility if the infection causes testicular damage such as disruption of the blood–testis barrier, an obstruction of the efferent seminal ducts or the formation of anti-sperm antibodies. This process is mostly independent of the pathogen. Therefore, considering that most of the public opinion and research efforts have been focussed on women’s and children’s health, and as men’s health has been neglected for several reasons, more research must be conducted in order to understand the long-term impact of sexually transmitted diseases on male reproductive functions.

## Supplementary Material

Supplemental Material
