# Supplementary material for: Long-term consequences of sexually transmitted infections on men’s sexual function: A systematic review
Source: Arab J Urol. 2021 Jul 7;19(3):411–8. doi: 10.1080/2090598X.2021.1942414 (PMC8451632; doi:10.1080/2090598X.2021.1942414)
Supplement: Supplemental Material [file TAJU_A_1942414_SM9741.docx]

**Supplementary Table 1:** Terms and phrases used for the PubMed search.

consequences AND "sexually transmitted infection" AND male sexual function

consequences AND "sti" AND male sexual function

"long term" AND "sti" male sexual function

"long term" AND "std" AND male sexual function

"long term" AND "sexually transmitted disease" AND male sexual function

"long term" AND "sexually transmitted infection" AND male sexual function

"long term" AND "sexually transmitted infection" AND sexual function

consequences AND "sti" AND sexual function

"long term" AND "sti" AND sexual function

consequences AND "sexually transmitted infection" AND sexual function

"long term" AND "chlamydia*" AND male sexual function

"long term" AND "mycoplas*" AND male sexual function

"long term" AND "neisse*" AND male sexual function

"long term" AND "coli*" AND male sexual function

"long term" AND "enterococ*" AND male sexual function

"long term" AND "staphyloco*" AND male sexual function

"consequenc*" AND "staphyloco*" AND male sexual function

"consequenc*" AND "enteroco*" AND male sexual function

"consequenc*" AND "coli*" AND male sexual function

"consequenc*" AND "neisse*" AND male sexual function

"consequenc*" AND "mycoplas*" AND male sexual function

"consequenc*" AND "chlamyd*" AND male sexual function

"effect" AND "chlamyd*" AND male sexual function

"effect" AND "neisse*" AND male sexual function

"effect" AND "mycoplas*" AND male sexual function

"effect" AND "coli*" AND male sexual function

"effect" AND "sexually transmitted" AND male sexual function

covid AND sperm

covid AND "male reproductive health"

"long term" AND "sexually transmitted disease" AND male reproductive health

consequenc* AND "sexually transmitted disease" AND male reproductive health

consequenc* AND chlamyd* AND male reproductive health

consequenc* AND neisse* AND male reproductive health

consequenc* AND mycoplas* AND male reproductive health
